# Soluble Transferrin Receptor during infancy and reference intervals for the Roche Cobas platform

**DOI:** 10.1111/ijlh.13391

**Published:** 2020-11-20

**Authors:** Sara Marie Larsson, Andreas Hillarp, Pia Karlsland Åkeson, Lena Hellström‐Westas, Magnus Domellöf, Ulrica Askelöf, Cecilia Götherström, Ola Andersson

**Affiliations:** ^1^ Department of Clinical Chemistry Hospital of Halland Halmstad Sweden; ^2^ Department of Clinical Sciences, Lund, Pediatrics Lund University Lund Sweden; ^3^ Department of Clinical Sciences Malmö, Preventive Pediatrics Lund University Lund Sweden; ^4^ Department of Women's and Children's Health Uppsala University Uppsala Sweden; ^5^ Department of Clinical Sciences, Pediatrics Umeå University Umeå Sweden; ^6^ Division of Obstetrics and Gynecology Department of Clinical Science, Intervention and Technology Karolinska Institutet Stockholm Sweden

**Keywords:** acute phase response, infancy, iron, reference intervals, sTfR

## Abstract

**Introduction:**

Infant iron status assessments may be difficult to interpret due to infections. The soluble transferrin receptor (sTfR) has been suggested as a biomarker mainly unaffected by the acute phase response. Reference intervals reflecting dynamics of infant growth first year in life are not well established.

**Methods:**

The sTfR and CRP concentrations were measured in samples from 451 term infants with the Roche Cobas platform in umbilical cord, at 48‐96 hours, 4 and 12 months. Reference values were constructed as the 2.5th and 97.5th percentiles. The relationship between CRP concentrations >1 mg/L and sTfR was tested by Kendall correlation.

**Results:**

Reference intervals for girls and boys were 2.4‐9.5 mg/L at birth, 2.9‐8.4 mg/L at 48‐96 hours, 2.6‐5.7 mg/L at 4 months and 3.0‐6.3 mg/L at 12 months. No differences between sexes were observed except for at 4 months. sTfR did not covariate with CRP concentrations >1 mg/L except in 48‐96 hours samples.

**Conclusion:**

This study reports reference intervals for sTfR from birth to 12 months of age in a large group of infants in a low‐risk area for iron deficiency. sTfR might add value to infant iron status diagnostics since no covariation with CRP was found at birth, at 4 months or at 12 months.

## 1 INTRODUCTION

Iron has important physiologic roles in early life; thereby assessment of infant iron status is relevant to healthcare worldwide. Iron status biomarkers are more or less affected by interplay with the acute phase response. As a crucial nutrient for many microorganisms, including pathogens such as the malaria *Plasmodium* parasite, human biochemical responses are developed to keep iron unavailable to infecting microorganisms.^1^

The soluble Transferrin Receptor (sTfR) has been suggested to be less influenced by the infectious and inflammatory status. Extensively studied, mainly in adults, several studies have emphasized its advantages in distinguishing between anemia of inflammation and iron deficiency anemia (IDA).^2^ It is generated by proteolytic cleavage of transmembrane dimeric glycoprotein transferrin receptor, as the red blood cell precursors mature; thus mainly shed from erythroblasts and reticulocytes. The relationship between tissue transferrin receptor and sTfR is reported to be constant, and sTfR by that reflects erythroid proliferation rate and iron turnover.^3^ Whereas ferritin is a marker of iron stores, sTfR has been described to be a marker of iron needs, which means that these two biomarkers do not necessarily correlate.^4^

Erythropoiesis as such is subject to large developmental changes subsequent to adaption to extrauterine life. Consequently, reference intervals need to be able to reflect the dynamics of infant growth. Furthermore, frequent infections complicate infant iron status assessments, but use of sTfR as a marker of iron status in infants has been questioned.^5^, ^6^

Although to the best of our knowledge, there are in total 14 studies of sTfR reference intervals during infancy published, only two of these refer to one of the most widely used commercial assays; Roche Diagnostics.^6^, ^7^, ^8^, ^9^, ^10^, ^11^, ^12^, ^13^, ^14^, ^15^, ^16^, ^17^, ^18^, ^19^, ^20^ Standardization has not been widely established and conversion of reference intervals between platforms is not possible.^16^ Age intervals vary across studies, and evaluation of published data is complicated. Moreover, previous studies have shown possible iron biomarker differences according to sex during infancy.^7^, ^21^

The aim of the present study was to calculate reference intervals, for the Roche Cobas sTfR assay, at four different time points in infancy and to investigate if partitioning according to sex is needed. A secondary aim was to describe sTfR in relation to increased C‐reactive protein (CRP) in infants born in a geographic area with low risk of iron deficiency and a low burden of infections.

## 2 MATERIALS AND METHODS

### 2.1 Study population

This was a retrospective longitudinal cohort study. The infants were born in a low‐risk area for iron deficiency, from uncomplicated pregnancies and uneventful perinatal circumstances and had a gestational age of 37^+0^‐41^+6^ weeks at delivery. The mothers were nonsmokers and healthy with uneventful pregnancies. Data were collected during 2008‐2015 as part of studies assessing timing of umbilical cord clamping.^22^, ^23^, ^24^, ^25^ The first study population comprised of two studies performed at the county hospital of Halland, one study with children born in vaginal births (n = 400) and one study with children born by elective cesarean section (n = 64). These were approved by the regional research ethics committee at Lund University (41/2008, 344/2009). Data were combined with an additional study population with infants born in vaginal births (n = 200) performed at a university hospital in Stockholm, Sweden. This study was approved by the regional ethical review board in Stockholm (2011/2142‐31/3). The parents declared subjective well‐being of their children.

Data from children delivered with cord clamping <30 seconds were excluded from reference interval calculations^26^ resulting in a dataset comprising 451 infants (Halland n = 252, Stockholm n = 199). In total, there were 214 boys and 237 girls. Results with a corresponding CRP of >10 mg/L were excluded from calculations. The total number of individuals coheres with the Clinical and Laboratory Standard Institute C28‐A3 guideline for establishing reference values with 90% confidence intervals.^27^

Of the infants in Halland, 64% had sTfR results and a corresponding CRP result at all four time points, 25% three time points, 10% two time points, and 1% at one time point. Data were mainly missing at 48‐96 hours due to increased tendency for hemolysis in the sample at this time point. Of the infants in Stockholm, 78% had sTfR results and a corresponding CRP at both two sampling time points.

Body weight at 12 months was within WHO Child growth standards^28^ except for two girls and nine boys who had slightly higher weights than the WHO 99th percentile (12.7, 13.5 kg and 12.5‐13.6 kg, respectively). Body lengths at 12 months were within WHO Child growth standards except for one girl (82 cm) and four boys (82‐83 cm) slightly above the WHO 99th percentile.

Since a clinical decision limit excluding iron deficiencies for this age group has been debated, we chose to illustrate robustness of the population by presenting reference values excluding individuals with ferritin concentrations below two arbitrarily set cutoff points (20 and 30 µg/mL, respectively).

### 2.2 Specimen collection and handling

Blood samples in studies performed at the hospital of Halland were taken at birth (umbilical cord blood), at 48‐96 hours in conjunction with the metabolic screening, at 4 months and at 12 months. For the children born in Halland, blood was collected in tubes with serum separator (BD Vacutainer, Plymouth, UK). Blood samples in the study carried out at Karolinska University Hospital were taken at birth (umbilical cord blood) and at 4 months and blood was collected in serum tubes with serum separator (Sarstedt AG & Co, Nümbrecht, Germany).

Blood sampling (a maximum of 2.5 mL blood) was at 4 and 12 months performed after application of a local dermal analgesia with lidocaine 2.5% and prilocaine 2.5% (EMLA, AstraZeneca).

### 2.3 Laboratory methodology

CRP, sTfR, and ferritin were analyzed using Cobas 6000 (Roche Diagnostics, Basel, Switzerland). Instruments were calibrated and run according to the manufacturer's instructions. The sTfR Tina‐quant (a particle enhanced immunoturbidimetric assay) was according to the manufacturer standardized to an internal Roche reference material. Total imprecision was estimated by use of a commercial internal control material from SERO AS (Billingstad, Norway). Calculated between‐day imprecision within the laboratory during 2008‐2015 was estimated to 4% at 2.0 mg/L and 5% at 3.4 mg/L.

The laboratory was accredited according to SE‐EN ISO/IEC 17025:2005 and participates in interlaboratory external proficiency testing schemes from Equalis AB (Uppsala, Sweden).

### 2.4 Statistical methodology

Data at each sampling time point were assessed separately. Data were mathematically transformed by the method of Box‐Cox. Transformation was ruled in as successful if a) the visual inspection of the Q‐Q‐plot was approved and b) if the hypothesis test of Anderson‐Darling passed with a significance level of 10%.

The 95% interpercentile reference interval was calculated for each group and a 90% confidence interval around each endpoint was estimated by using biweight quantile, transformed, reflected methodology with 500 bootstrap samples. Differences between time points and sexes were assessed as significant if 90% confidence intervals were nonoverlapping.

Relation between sTfR and CRP in samples with a CRP result >1 mg/L was assessed with Kendall rank correlation as data were non‐normally distributed.

Statistical analyzes were conducted using Analyse‐it for microsoft excel 4.90.4 from Analyse‐it Software Ltd.

## 3 RESULTS

### 3.1 Reference intervals

**Figure 1:**
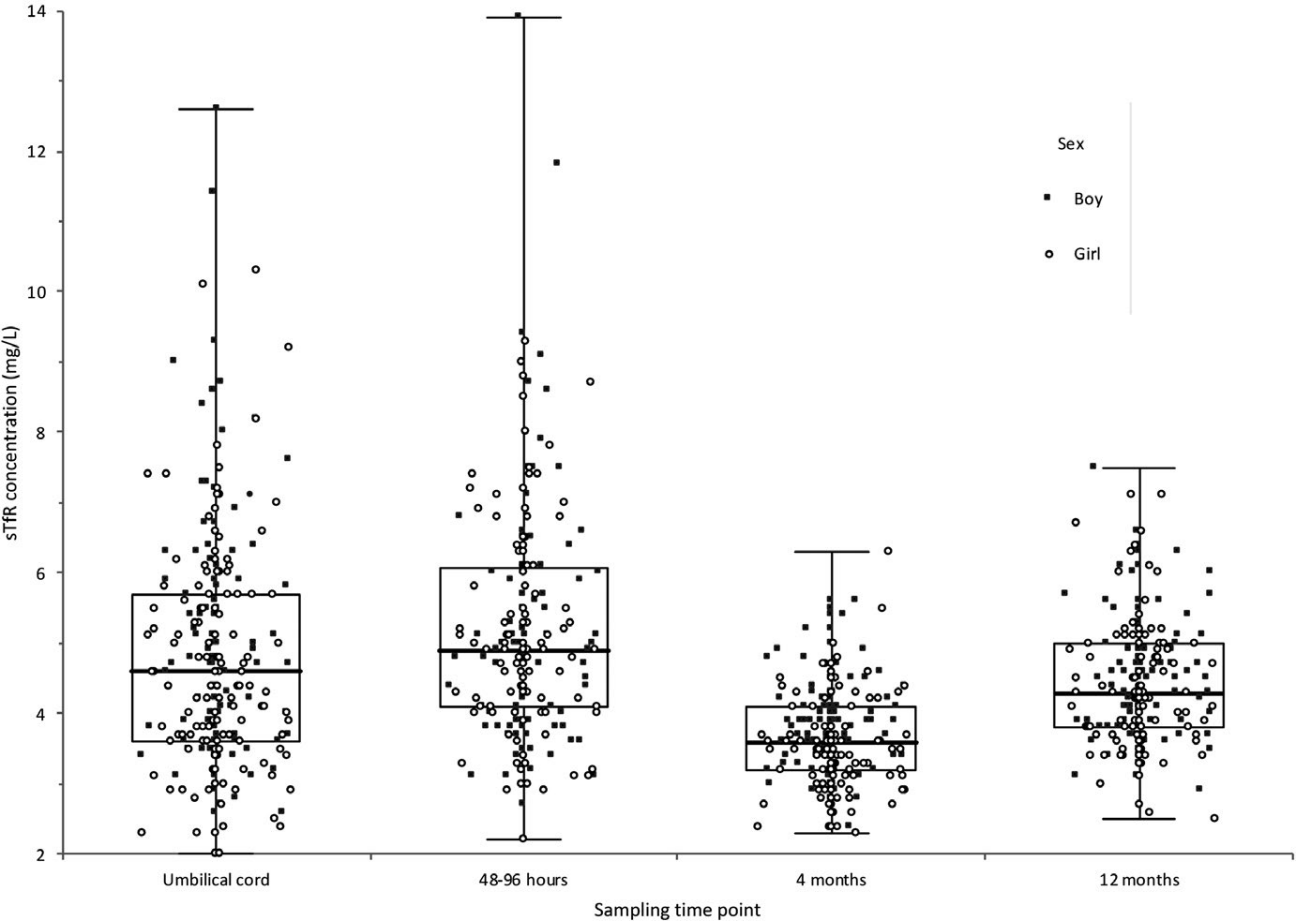
Data distribution (nontransformed) of soluble Transferrin Receptor (sTfR) concentrations (mg/L) at the four different sampling time points illustrated by bee‐swarm box and whiskers plots

Distribution of the original sTfR concentration data at the four sampling time points is shown by bee‐swarm box plots in Figure 1. Lower and upper reference values with 90% confidence limits partitioned by age and the two suggested cutoffs for ferritin are shown in Table 1.

**Table 1:** Reference intervals for sTfR (mg/L) as the 2.5th and 97.5th percentiles calculated by Box‐Cox transformation

Sampling time point	n	Ferritin cutoff	sTfR concentration (mg/L)			
			**Lower (2.5th percentile)**	Lower 90% CI	**Upper (97.5th percentile** **)**	Upper 90% CI
Umbilical cord	438	None	2.4	2.3‐2.5	9.5	8.8‐10.4
48‐96 h	165	None	2.9	2.7‐3.0	8.4	7.8‐8.9
4 mo	373	None	2.6	2.5‐2.6	5.7	5.4‐5.9
	366	≥20 µg/L	2.6	2.5‐2.7	5.6	5.4‐5.9
	356	≥30 µg/L	2.6	2.5‐2.6	5.5	5.3‐5.8
12 mo	191	None	3.0	2.9‐3.1	6.3	6.1‐6.6
	167	≥20 µg/L	2.9	2.8‐3.0	6.1	5.8‐6.3
	127	≥30 µg/L	2.9	2.7‐3.0	6.1	5.9‐6.5

Note

Inclusion of individuals at two arbitrarily set ferritin cutoffs 20 and 30 µg/L, respectively; in comparison with no ferritin cutoff applied to calculations.

#### 3.1.1 Blood sampling from umbilical cord

The lower reference value of sTfR was slightly higher in boys than in girls and lower 90% confidence intervals were nonoverlapping. Corresponding upper confidence interval pointed to no differences between sexes.

#### 3.1.2 Blood sampling at 48‐96 hours

We found no significant differences in sTfR according to sex at 48‐96 hours, neither at the lower nor at the upper reference levels.

#### 3.1.3 Blood sampling at 4 months

Differences in sTfR according to sex were most pronounced at 4 months and reference values of boys were higher than those of girls. Confidence intervals marginally overlapped and pointed at possible differences between the sexes. Lower reference values at 4 months were slightly higher compared with the umbilical cord values. The upper reference value was approximately 40% lower compared with values in umbilical cord.

#### 3.1.4 Blood sampling at 12 months

At 12 months, the confidence intervals of sTfR in girls and boys were nonoverlapping at the lower reference value and the upper reference value was approximately the same for boys and girls with almost completely overlapping confidence intervals.

With regard to age partitioning, there were significant differences between all four time points during the first year of life, indicating that sTfR follows the dynamic changes of infant growth.

The 90% confidence intervals surrounding the 2.5th and 97.5th percentiles did overlap for the reference values calculated with the arbitrarily set ferritin cutoff points, pointing to no significant differences in sTfR reference values in this ferritin concentration span, Table 1.

### 3.2 Association to CRP as a marker of the acute phase response

**Figure 2:**
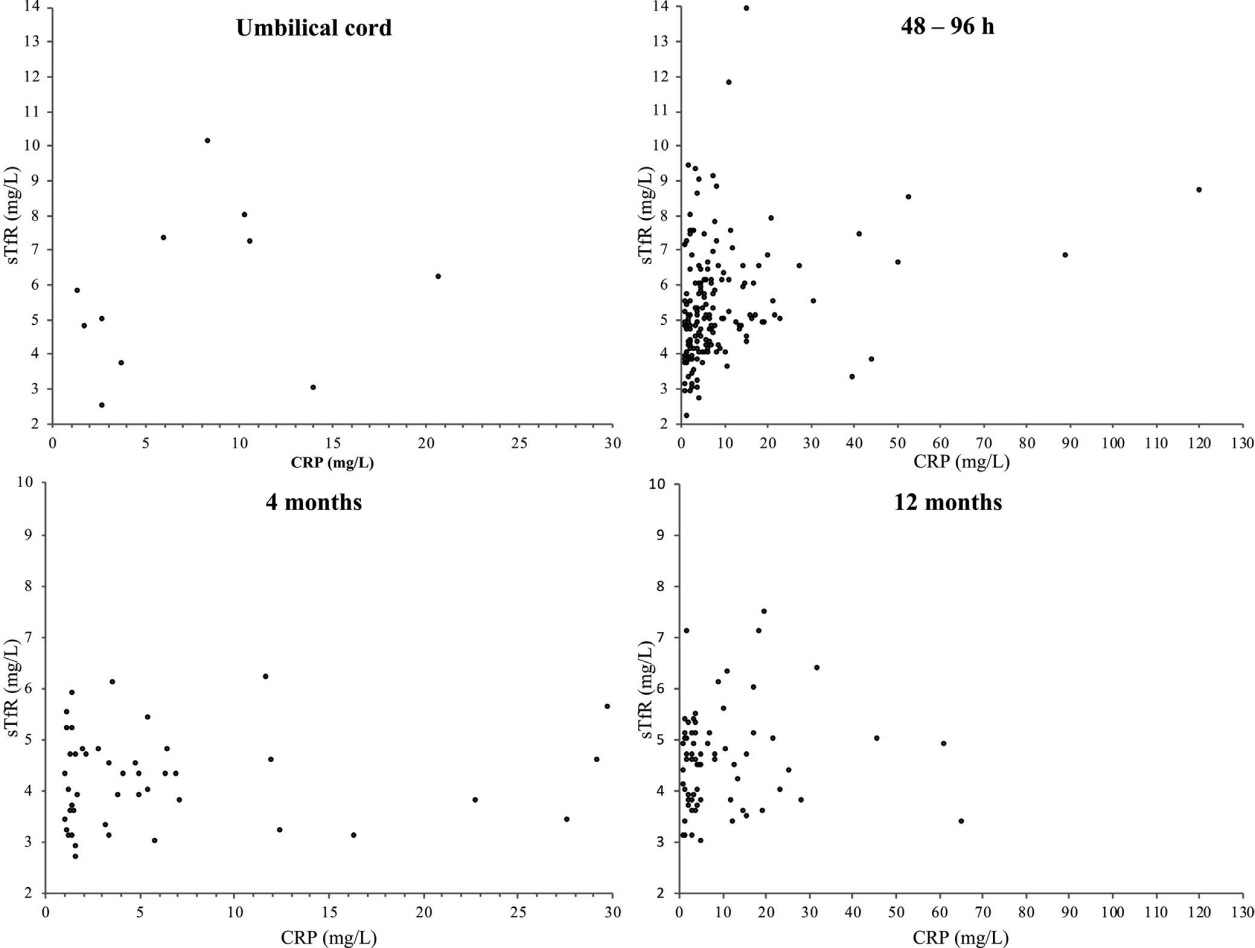
Scatterplots of the CRP concentrations >1.0 mg/L vs corresponding sTfR concentrations (mg/L) at the 4 sampling time points

At 48‐96 hours, a correlation between an increased CRP concentration and sTfR was observed τ_48‐96 hours_ = 0.20 [0.09, 0.30] *P* < .0001 (n = 172). In the umbilical cord, at 4 months or 12 months there was no observed correlation τ_umbilical cord_ = 0.15 [−0.26, 0.55], *P* = .53 (n = 11); τ_4 months_ = 0.04 [−0.18, 0.25], *P* = .73, (n = 45) and τ_12 months_ = 0.08 [−0.09, 0.26], *P* = .33, (n = 68). Scatter plots are presented in Figure 2.

## 4 DISCUSSION

The sTfR concentration has been suggested as an iron status biomarker in the pediatric population. There are many factors associated with normal growth that may influence interpretation of sTfR results in medical decision‐making and reference intervals also considering changes during the first year of life have been scarce. In this study, we present reference intervals for sTfR at four different time points from birth to 12 months of age in a well‐defined large cohort of healthy infants with methodological references to Clinical and Laboratory Standards Institute CLSI guidelines^27^ for the commonly used Roche Cobas platform.

Previously published reference intervals were compiled as listed in Table 2. Altogether possibility for comparison, for trends as well as actual reference values, was limited due to generally small study populations, variably summarized reference intervals, different assay methodology, or lack of assay methodology information and use of parametric statistical methods when describing data not normally distributed. Also, some intervals were calculated with extrapolated data from populations mainly covering older infant ages, thus potentially missing important developmental phases of earlier ages.

**Table 2:** Review of previously published reference intervals (1997‐2009)

Authors ^reference^ (y) Country	Sample size Infant age	Results sTfR (mg/L)	sTfR assay according to published article	Reported as according to published article
Yeung and Zlotkin^18^ (1997) Canada	8.6‐15.2 mo (n = 485) 9 mo (n = 24) 10 mo (n = 60) 11 mo (n = 70) 12 mo (n = 90)	2.9‐4.5 2.9‐6.5 2.9‐6.5 3.2‐6.7	Indirect enzyme‐linked immunosorbent assay. Standard used TfR isolated from human placental tissue. Primary monoclonal antibody against human TfR was supplied by Cetus/Chiron (clone 454A12: Emeryville, CA): Secondary goat antibody by Calbiochem, SanDiego).	Graphical 5th and 95th percentile estimates
Olivares et al^10^ (2000) Chile	8‐15 mo (n = 515) resulting in 716 samples. 8 mo (n = 51) 12 mo (n = 54)	6.3‐13.2 5.9‐13.8	2‐site enzyme‐linked immunoassay with monoclonal antibodies prepared against sTfR purified from human placenta.	Median with 95% CI
Kling et al^8^ (1998) USA	1‐7 mo (n = 22) 1 mo 2 mo 3 mo 4 mo 5 mo 6 mo 7 mo	1.38‐2.06 2.02‐2.50 ‐0.95‐5,73 2.23‐3.01 2.22‐2.96 2.13‐2.89 2.17‐2.97	Quantikine^™^ IVD Human TfR Immunoassay kit (R&D Systems, Minneapolis, USA)	Mean ± SD
Kratovil et al^9^ (2007) USA	6‐24 mo (n = 37) Leftover complete blood count samples from patients with normal Hb, Hct, MCV and MCHC for age.	1.37‐2.85	Quantikine^™^ IVD Human TfR Immunoassay kit (R&D Systems, Minneapolis, USA)	2.5th‐97.5th percentile
Vazquez Lopez et al^15^ (2006) Spain	12 mo‐10 y (n = 368) 206 subjects with normal iron status	1.52‐2.34	Quantikine^™^ IVD Human TfR Immunoassay kit (R&D Systems, Minneapolis, USA)	Media ± SD
Kuiper‐Kramer et al^6^ (1998) The Netherlands	Healthy infants, gestational age of 34 wk or more born after uncomplicated labor. (n = 98) Umbilical cord	6.30‐14.63	Ramco transferrin receptor assay, purchased from DPC, Apeldoorn, The Netherlands. Microplate absorption photometer type ht, Anthos, Austria.	10th‐90th percentile
Domellöf et al^20^, ^21^ (2002) Sweden	Unselected normative population approach; population likely to have a low prevalence of iron deficiency. 4 mo (n = 197) 6 mo (n = 134) 9 mo (n = 71)	2.2‐11.0 2.7‐11.5 2.5‐15.0	TfR (Ramco, Houston, Tex)	Graphical estimates of mean ± 2SD
Virtanen et al^17^ (1999) Finland	12 mo (n = 36)	4.5‐11.1	Enzyme‐linked immunosorbent assay Ramco Laboratories, Inc, Houston	95% reference interval
Suominen et al^13^ (2001) Finland	6 mo‐4 y (n = 52) Elective surgery (short term) Approximately 2‐3 children <12 mo (graphical illustration)	1.5‐3.3	Immunoturbidimetric assay (IDeA^®^ sTfR‐IT Orion Diagnostica) on a Hitachi 917 analyzer	2.5th and 97.5th reference limits with 95% CI around each limit
Schiza et al^12^ (2007) Greece	Preterm (32‐36 w gestational age) (n = 188) 2 wk 6 wk 3 mo 6 mo 9 mo 12 mo	1.5‐3.5 1.3‐5.3 2.1‐6.3 2.7‐4.7 2.9‐5.1 2.8‐6.2	Noncompetitive “sandwich type” enzyme‐immunoassay technique, using monoclonal antibody (Orion Diagnostica, Espoo, Finland)	Mean (±SD? Not reported)
Hay et al^7^ (2007) Norway	Healthy, term infants (n = 350) Umbilical cord (n = 350)	3.8‐14.9	IDeA sTfR IEMA assay; Orion Diagnostica, Turku, Finland.	5th‐95th percentile reference intervals
Takala et al^14^ (2009) Finland	Full‐term (>37 wk) (n = 50) 1‐7 d after birth 5 mo (20 wk) 6 mo (25 wk) 8 mo (30 wk) 9 mo (35 wk) 10 mo (40 wk) 11 mo (45 wk) 12 mo (50 wk)	1.29‐3.29 1.06‐2.08 1.08‐2.12 1.09‐2.19 1.09‐2.27 1.08‐2.37 1.07‐2.48 1.05‐2.62	sTfR assays were performed with a commercial automated immunoturbidimetric method (IDeA sTfR‐IT; Orion Diagnostica, Espoo, Finland) using the Modular P analyzer (Roche Diagnostics, Basel, Switzerland).	95% reference interval
Vendt et al^16^ (2009) Estonia	9‐12 mo (n = 179) N = 146 (IDeA^®^) N = 80 (Tina‐quant^®^)	1.5‐2.7 4.1‐7.8	Immunoturbidimetric method (IDeA^®^ sTfR‐IT; Orion Diagnostica, Espoo, Finland) on the analyzer Cobas Mira (ABX Diagnostics, Basel, Switzerland) and Tina‐quant^®^ Soluble Transferrin Receptor (Roche Diagnostics GmbH, Mannheim, Germany) on the analyser Cobas Integra 400 (Roche Diagnostics GmbH)	5th and 95th percentile with 95% CI around each limit
Heiduk et al^29^ (2009) Germany	0‐1 y (n = 95) (a posteriori approach, reference population carefully selected from ambulatory and hospitilized children according to clinical status and chemical profile).	1.55‐5.7	sTfR latex enhanced immunoturbidimetry Tina‐quant (Roche Diagnostics) on analyser Modular Analytics (Roche Diagnostics, Mannheim, Germany)	2.5th and 97.5th percentiles

Note

Statistical data and laboratory methodological information as presented in corresponding article.

Due to the standardization issues, results from our study could only be compared with those two using the same assay platform; by Heiduk et al^29^ and by Vendt et al^16^ The study by Heiduk et al was based on ambulatory and hospitalized children selected according to clinical status and chemical profile, while our population is based on a community‐based cohort of presumably healthy infants. Also, their study was based on an age partitioning of 0‐1 year thereby not describing differences in sTfR concentrations within this time frame.^29^ Our data were in coherence at 4 months. At 12 months, Heiduk et al reported a lower upper reference value compared with our study. The other study, by Vendt et al, covered ages 9‐12 months. Comparing data at 12 months, they reported the 5th percentile as well as the 97.5th percentile to be higher.^16^ Differences may be due to study populations or bias between instruments/laboratories as well as statistical methodology used describing data.

We also compared the reference intervals in this study to those previously published for adults for the Roche Cobas assay; for women ≥ 17 years 1.9‐4.4 mg/L and for adult men ≥ 17 years 2.2‐5.0 mg/L.^30^ In coherence to findings by Kuiper‐Kramer et al^6^ reference values in our study were higher compared to adults. The constant relationship between sTfR levels and its membrane form would ideally need to be validated also in early infancy. Thus, adult reference values should not be used for infants and care has to be taken when correlating sTfR to iron metabolism.

The lack of sTfR assay standardization has contributed to a limited usability of this biomarker in iron status assessments and notably, comparisons between assays have shown poor agreement.^16^ The 1st WHO reference reagent preparation 07/202 was made available in 2010 to improve the situation.^31^ However, although available for a decade, reagent preparation 07/202 has to the best of our knowledge not been widely established as a reference material in commercial in‐vitro diagnostics reagent kits on the market, including the one by Roche Diagnostics that is used in this study. This calls for further investigations and until then, assay‐specific reference intervals are still needed.

In this study, we also set out to investigate associations of acute phase response, as measured by CRP, with sTfR. Studies in children with inflammatory conditions and infectious diseases are currently few and with contradictory results.^11^, ^32^, ^33^ Similarly to conclusions by Rohner et al,^32^ our data did not show sTfR to be associated with the acute phase response as assessed by CRP at 4 months or 12 months. There was, however, a significant association between sTfR and CRP at 48‐96 hours which may be due to an increase in transferrin receptor expression by hypoxia induced by uterine contractions during normal labor. Intrauterine pressure transiently compromises oxygenation, but most healthy, term fetuses are able to withstand as there are several fetal defenses against hypoxic injury.^34^ As the same processes activate inflammatory cytokine pathways during normal labor this might be the reason why a covariation between CRP and sTfR was observed.

A major strength of our study is the longitudinal design and data collection of blood samples from 451 healthy infants from uncomplicated pregnancies in an area with low risk for iron deficiency. Parents declared subjective well‐being of the child, but we cannot fully exclude the possibility of a child having some underlying condition. What criteria that with certainty could serve diagnosing infant iron deficiency is still a subject of discussion. Our study may have included some infants with iron deficiency. The reliability of our data was therefore tested by excluding infants with a corresponding ferritin <20 µg/L and <30 µg/L. The sTfR reference values at 4 and 12 months were not subject to significant changes by this partitioning. It is therefore unlikely that these sTfR reference values are affected by iron deficiency. Another concern is that the reference values could be affected by hematological disorders, such as thalassemia or hemochromatosis. However, prevalence of thalassemia is very low in northern Europe^35^ and juvenile hemochromatosis is very rare.^36^ Also, results could be affected by the timing of umbilical cord clamping. In this study, a minimum of 30 seconds was selected; based on the American College of Obstetricians and Gynecologists committee opinion,^26^ but this time of clamping delay can be regarded as short as WHO recommendation is to delay cord clamping and cutting for 1‐3 minutes for term infants.^37^

A weakness of our study is that the approach used cannot establish decision limits, and the intention of our study is not that the 97.5th percentile should be used as such. To establish decision limits, studies need to be performed with a design based on clinical adverse outcomes which is not possible to perform ethically. Reference intervals do not imply a decision limit, but should be considered as a statistical model describing the distribution of a biomarker for a well‐defined population. Also, the reference intervals calculated in this study are specific for the Roche Cobas assay and traceability to 1st WHO reference reagent preparation 07/202 is lacking. Another weakness is the few numbers of elevated CRP concentrations in the umbilical cord samples used to estimate the nonparametric correlations with sTfR.

In conclusion, this study present reference values for the iron status biomarker sTfR in a large cohort of presumably healthy infants measured on the Roche Cobas platform. The biomarker sTfR might add value to infant iron status diagnostics since no covariation with CRP was found at birth, at 4 months or at 12 months.

## CONFLICT OF INTEREST

The authors report no conflicts of interest.

## AUTHOR CONTRIBUTIONS

Andersson, Hellström‐Westas, and Domellöf involved in *study concept and design*. Andersson, Askelöf, and Götherström involved in *acquisition of data*. Larsson and Andersson involved in *analysis and interpretation of data*. Larsson, Andersson, Hillarp, and Karlsland Åkeson involved in *drafting of the manuscript*. Andersson, Hillarp, Karlsland Åkeson, Hellström‐Westas, Domellöf, Askelöf, and Götherström involved in *critical revision of the manuscript for important intellectual content*. Larsson and Andersson involved in *statistical analysis*. Larsson, Hillarp, Karlsland Åkeson, and Andersson *obtained funding*.

## Data Availability

The data that support the findings of this study are available on request from the corresponding author. The data are not publicly available due to privacy or ethical restrictions.
